# Relationship of a Special Acidified Milk Protein Drink with Cognitive Performance: A Randomized, Double-Blind, Placebo-Controlled, Crossover Study in Healthy Young Adults

**DOI:** 10.3390/nu10050574

**Published:** 2018-05-08

**Authors:** Yoshie Saito, Natsuko Murata, Teruyuki Noma, Hiroyuki Itoh, Mitsunori Kayano, Kimihide Nakamura, Tadasu Urashima

**Affiliations:** 1 R & D Division, Meiji Co., Ltd., Tokyo 192-0919, Japan; natsuko.murata@meiji.com (N.M.); teruyuki.noma@meiji.com (T.N.); hiroyuki.itou@meiji.com (H.I.); 2 Research Center for Global Agromedicine, Obihiro University of Agriculture and Veterinary Medicine, Hokkaido 080-8555, Japan; kayano@obihiro.ac.jp; 3 Health Care Administration Center, Obihiro University of Agriculture and Veterinary Medicine, Hokkaido 080-8555, Japan; k.nakamura@zhi.or.jp; 4 Obihiro Dai-ichi Hospital, Hokkaido 080-0014, Japan; 5 Graduate School of Food Hygiene, Obihiro University of Agriculture and Veterinary Medicine, Hokkaido 080-8555, Japan; urashima@obihiro.ac.jp

**Keywords:** milk protein, cognitive performance, heart rate variability, clinical study

## Abstract

A previous in vivo study with rats suggested that a special milk protein drink manufactured using an acidification procedure to suppress the aggregation of milk proteins was absorbed quickly after feeding. We performed a randomized, double-blind, placebo-controlled, repeated-measure crossover study to investigate the short-term effects on cognitive performance in 29 healthy young adult men after they consumed this drink in the morning. After an overnight fast, subjects were tested for performance in the Uchida–Kraepelin serial arithmetic test and the Stroop test as well as for subjective feeling, body temperature, and heart rate variability before and after consumption of either the acidified milk protein drink or an isoenergetic placebo drink. Subjects showed a significant improvement in performance in the Uchida–Kraepelin test, the primary outcome measured, when they consumed the acidified milk protein drink compared with the placebo control condition. In addition, consumption of the acidified milk protein drink, compared with the placebo control, was associated with increases in vagally-mediated heart rate variability indices which, from recent theoretical perspectives, may reflect a higher ability to modulate cognitive and behavioral processes. There was no significant difference in subjective feelings and body temperature between the test drink conditions. These data suggest that consumption of the acidified milk protein drink may improve cognitive performance, with possible involvement of physiological systems that regulate cognition and behavior.

## 1. Introduction

Breakfast plays an important role in supplying energy and nutrients [1], yet breakfast skipping and selection of foods of low nutritional quality are common, especially in urban students [2]. In addition to nutritional benefits, there is growing evidence that breakfast may enhance cognitive and academic performance [3,4]. To obtain effective nutritional strategy, it is important to clarify the contributions of different macronutrients in breakfast on cognitive performance. Glucose is the main fuel for brain function. Intake of carbohydrates has a direct effect on blood glucose levels and, in turn, on cognitive function [5,6]. Both long- [7,8] and short-term [9] effects of consumption of specific kinds of fats on cognitive function have also been demonstrated. As to protein, prior works have demonstrated both long- [10,11] and short-term [12,13] effects of consumption of high-protein meal on cognitive performance compared to an isoenergetic high-carbohydrate meal. However, in the studies investigating the acute effects of protein on cognitive performance, the protein content consumed was much higher than that indicated in the Recommended Dietary Allowance (RDA). Even though the RDA recommends 0.8 g protein per kg body weight per day in the United States [14] and 60 g per day for males older than 12 years in Japan [15], the high-protein meals used in these studies contained 80–100 g per meal. The effect of adequate amounts of protein within a single meal on cognitive performance needs to be clarified.

Dairy products are good source of protein. Frequent dairy product consumption is found to be associated with better cognitive performance [16,17], although the underlying mechanism is still to be determined. An acute beneficial effect of dairy milk on cognition is also reported in persons with higher fasting glucose [18]. It is well recognized that the protein fraction in bovine milk contains a balanced profile of essential amino acids and is highly digestible, and its amino acids are highly absorbable [19,20]. However, absorption of the components in bovine milk is slowed by gastric acid—induced coagulation of caseins, which constitute up to 80% of the milk protein fraction [21]. To suppress this aggregation, an acidification procedure for milk has been developed using stabilizers as well as a dispersant [22]. Amino acids from the acidified milk appeared to be absorbed more quickly compared with untreated milk, as was shown by a greater increase in the plasma amino acid level after ingestion in rats.

A mental arithmetic task is one of the tasks to assess cognitive performance. Higuchi demonstrated that nutritionally balanced breakfast improved the task performance of the paper-based mental arithmetic task compared to a high-carbohydrate breakfast [23]. Certain physiological indices may be associated with cognitive performance [24,25,26]. One such index is body temperature. Within an optimal thermal range, increased body temperature has been associated with improved cognitive performance and alertness in humans, as shown by studies in which the circadian phase was controlled [24,25]. Another index that may be associated with cognitive performance is heart rate variability (HRV). HRV is the fluctuation in the beat-to-beat interval in the heart rate, resulting from the interaction between the sympathetic and parasympathetic arms of the autonomic nervous system [27]. It has been suggested that HRV reflects the capacity of individuals to respond to changing environmental demands, and it has often been considered to be a biomarker of the ability to regulate cognitive, emotional and behavioral processes [26].

We hypothesized that a special milk protein drink may have an acute beneficial effect on cognition and modify associated physiological indices. To test this, we performed a crossover study to investigate the acute effects of an acidified milk protein drink on cognitive performance as well as subjective feeling, body temperature, and HRV compared to an isoenergetic placebo drink.

## 2. Materials and Methods

### 2.1. Study Design

We conducted a randomized, double-blind, placebo-controlled, repeated-measure crossover trial at Obihiro University of Agriculture and Veterinary Medicine, Hokkaido, Japan, from 2–31 August 2016. The study protocol was approved by the Obihiro University of Agriculture and Veterinary Medicine Institutional Review Board of Clinical Research and the Meiji Institutional Review Board. Each subject provided written informed consent before randomization. The protocol was registered in the UMIN Clinical Trials Registry (UMIN000023373). A data-monitoring committee assured accuracy of data collection and inputs. All of the study procedures including explanation, written informed consent acquisition, questionnaire and testing were conducted in Japanese.

### 2.2. Subjects

Healthy male students of Obihiro University of Agriculture and Veterinary Medicine aged 18 to 30 years old were eligible to participate in this study. Only men were examined in this protocol because, although evidence at present is limited, it is suggested that cognitive function is modulated by the menstrual cycle in women [28]. The exclusion criteria were (1) milk or soy allergies; (2) serious or progressive illness such as cancer and hepatitis; (3) difficulty in distinguishing between reds, blues, greens and yellows; (4) regularly taking drugs, quasi drugs (a category of products with mild effects on the body), health foods or supplements that may affect this study within 3 months prior to the start of this study; (5) participation in other clinical studies within 1 month prior to the start of this study; (6) judged as ineligible by the medical doctor or lead principal investigator for other reasons. The presence of exclusionary criteria was determined by self-reporting in the questionnaire about health status and lifestyle.

### 2.3. Test Drinks

Subjects consumed 430 mL of either an acidified milk protein drink or a placebo drink that had a similar taste and appearance. Subjects were tested in 2 sessions separated by more than 2 days. Subjects in group 1 consumed the acidified milk protein drink at the first session and a placebo drink at the second session. Subjects in group 2 consumed the test drinks in the opposite order. Table 1 shows the composition of each test drink. The acidification procedure was based on a procedure developed by Nakayama et al. [22]. The drinks were manufactured by the R&D Division in Meiji Co., Ltd. (Kanagawa, Japan).

### 2.4. Study Procedure

The performance of the Uchida–Kraepelin test (UKT), a paper based serial mental arithmetic task, was designated as the primary outcome measure based on the previous study examining the acute effect of breakfast. The secondary outcome measurements were the Stroop test performance, subjective feelings, body temperature and HRV.

To minimize the practice effect, subjects practiced all cognitive tasks and the subjective feeling questionnaire before the study period. During the study period, subjects were required to refrain from changing their living and dietary habits and to report any non-routine consumption of drugs, quasi drugs, health foods or supplements. Subjects were not allowed to consume alcohol or to exercise excessively on the day before the testing. Subjects were also asked to refrain from consuming any food or drink other than water after 22:00 and to sleep before 24:00. Subjects were not allowed to smoke on the testing day.

**Figure 1:**
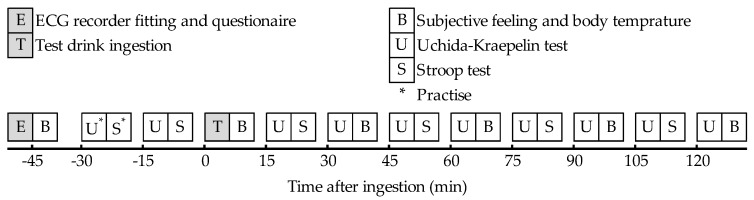
Schedule of the test session. After the fitting of the electrocardiogram (ECG) recorder and taking the compliance questionnaire, subjects underwent a baseline assessment: they recorded their subjective feelings and had their body temperature taken, practiced taking the Uchida–Kraepelin test (UKT) and the Stroop test, then they took the UKT and the Stroop test. Each subject then ingested the test drink, and repeated postprandial measurements were taken within the next 120 min. ECG was recorded throughout the test session.

The timing of the tests was decided based on the previous study [23]. Figure 1 shows the schedule of the test session. Subjects arrived at the laboratory room around 8:45. Just after arrival, subjects were attached to the electrocardiogram (ECG) recorder. They then filled out a questionnaire checking their compliance with the restrictions. Subjects then underwent baseline assessment. Subjects practiced the UKT and the Stroop test once before the baseline measurement, and then consumed the test drink within 5 min. Repeated postprandial measurements were taken within the next 120 min. ECG was recorded throughout the test session.

### 2.5. Psychological Measurement

#### 2.5.1. Uchida–Kraepelin Test (UKT)

The UKT (Nisseiken, Tokyo, Japan), a serial mental arithmetic task, was used to measure cognitive performance. The test material includes a pre-printed paper containing 34 rows of 115 random, single-digit figures. The subjects were asked to add adjacent figures horizontally, write the one digit of the answer between the 2 figures, and proceed along each row as fast and as accurately as possible in a 1-min period. When the first cue was given, subjects started calculating from the first row. After 1 min, the second cue was given and the subjects began on a new row regardless of their position on the current row. This was repeated 3 more times. The number of correct answers for the 1-min period was obtained, and the median value among the 5 times was evaluated.

#### 2.5.2. Stroop Test

The New Stroop Test II (Toyo Physical, Fukuoka, Japan) was used. The test material comprised pre-printed sheets consisting of 4 tasks. Each task included 100 questions, and subjects were asked to answer them as quickly and as accurately as possible within a 1-min period. Any subject who answered all of the questions within a 1-min period in any task was excluded from the analysis because maximal performance could not be measured. The number of correct answers (CA) for each task, the Stroop interference ratio ((task 3 CA − task 4 CA)/task 3 CA × 100), and the reverse Stroop interference ratio ((task 1 CA − task 2 CA)/task 1 CA × 100) were evaluated.

#### 2.5.3. Subjective Feeling

Subjects were instructed to fill in five 100-mm visual analog scales (VAS) to measure their sensations of hunger, fatigue, concentration, heat and sleepiness. Each VAS had a 0 endpoint indicating “do not feel at all” and a 100 endpoint indicating “strongly feel”. Subjects were asked to mark the location on the line that represented their mood at the time. The distance from the 0 end point to the mark was measured in mm.

### 2.6. Physiological Measurements

#### 2.6.1. Heart Rate Variability (HRV)

The interbeat interval (IBI) from the ECG was assessed using the ActiHeart recorder (CamNtech Ltd., Cambridge, UK). The recorder was attached just below the apex of the sternum while another end was attached to the left side and kept horizontally straight, using 2 ECG electrodes (SMA-150, METS Inc., Tokyo, Japan). The R-waves as well as the signal quality were recorded with the short-term recording mode in 15-s epochs.

HRV indices were calculated using the ActiHeart Software version 4.0.127 with 1-min analysis epochs. The time domain indices were the root mean squared successive differences between the IBIs (RMSSD) and the standard deviation of the IBIs. The frequency domain HRV indices were low-frequency power (LF), high-frequency power (HF), and the ratio between the LF and HF components (LF/HF). The 4-min time period during UKT, beginning at 30 to 45 s after the subject began the UKT depending on the recording epoch, were selected for analysis. The subjects with invalid data or data with a signal quality lower than 0.8 were excluded, according to the manufacturer’s recommendation. The average value of the 4-min time period was evaluated.

#### 2.6.2. Body Temperature

Oral temperature was measured by electronic thermometer (MC-672L, Omron, Kyoto, Japan). Subjects put the thermometers under their tongues for 5 min and terminal temperatures were obtained.

### 2.7. Statistical Analysis

Statistical analyses were conducted using BellCurve for Excel (SSRI Inc., Tokyo, Japan). We hypothesized that a milk protein drink may have an acute postprandial effect on cognition in crossover design. To assess the postprandial effect of the drinks throughout the testing time, the net incremental area under the curve (niAUC), calculated from the pre- and postprandial time points, were tested. The preprandial time point was used as the 0-min time point for niAUC calculations. In the crossover trial, the period effects, such as familiarization with the study situation, must be taken into an account. In the two periods design, this is achieved by testing the within-subject differences between both periods by t-tests or, in the case of non-normal distribution, non-parametric tests [29,30]. First, to check the assumption of negligibility of residual effects, the sum of the values in the two periods is calculated for each subject and compared across the two groups. Then, in case of no significance, treatment effects were assessed for the within-subject difference in the values between the two periods. Mann–Whitney U tests with continuity correction were carried out in this study because of the non-normal distribution. For all analyses, *p* values of less than 0.05 were considered statistically significant. An effect size (*r*) was calculated by dividing *z* by the square root of the total sample size across both groups [31]. Cohen’s guidelines indicate *r* = 0.10 is a small effect; *r* = 0.30 is a medium effect; and *r* = 0.50 is large effect.

## 3. Results

**Figure 2:**
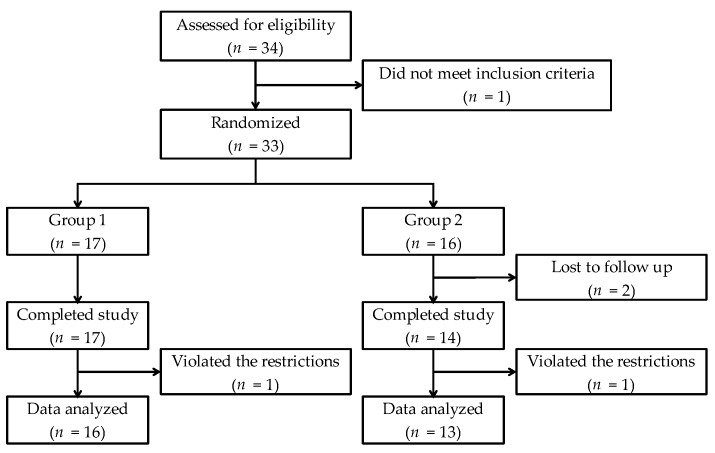
Flow of subjects through the study.

Figure 2 shows the flow of the subjects through the study. Twenty-nine subjects aged 19 to 24 years old (mean age 21.4 years, standard deviation 1.3 years), excluding 2 subjects who violated the restriction (the one who consumed food in the morning on the testing day, and the one who exercised excessively on the night before the testing day), were statistically analyzed. No adverse reactions were reported.

There were no significant residual effects for any of the analyses, indicating that the effect of test drinks given in one period did not affect the outcomes of the following period.

### 3.1. Psychological Measurements

**Figure 3:**
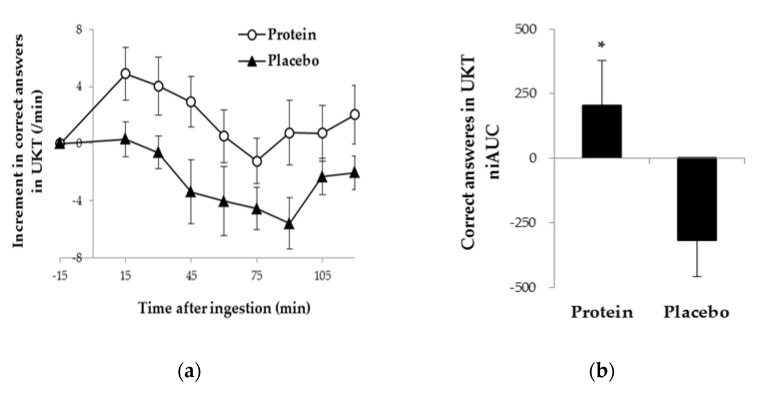
The numbers of correct answers in the UKT in protein or placebo drink condition: (**a**) increments from the baseline; (**b**) the net incremental area under the curve (niAUC). Data are represented as means ± standard errors. * Significantly different from Placebo, *p* < 0.05. *n* = 29.

Figure 3 shows the increments from the baseline values and the niAUC of the numbers of UKT correct answers. The niAUC was significantly higher when subjects consumed the acidified milk protein drink compared to the placebo drink (*p* = 0.044). Medium effect size was found in favor of the acidified milk protein drink condition (*r* = 0.37). For the Stroop test, 25 subjects, excluding 4 subjects who answered all of the questions within 1 min, were analyzed. There was no significant difference in the niAUC of the number of correct answers for each task, Stroop interference ratio, and reverse Stroop interference ratio, between the test drink conditions. Subjective feelings were similar between the test drink conditions.

### 3.2. Physiological Measurements

**Figure 4:**
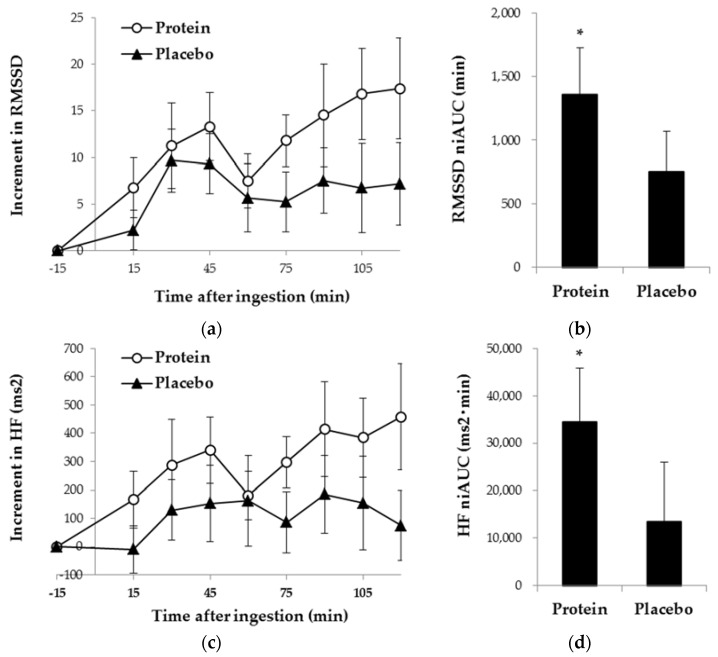
Heart rate variability (HRV) indices during the UKT in protein or placebo drink condition: (**a**) increment from the baseline of root mean squared successive differences (RMSSD); (**b**) the niAUC of RMSSD; (**c**) increment from the baseline of high-frequency power (HF); (**d**) the niAUC of HF. Data are represented as means ± standard errors. * Significantly different from Placebo, *p* < 0.05. *n* = 19.

The HRV data of 6 subjects were lost due to an error in the data recording. Four subjects were excluded from the data analysis because they had either invalid data or data with a signal quality lower than 0.8. Thus, 19 subjects were analyzed for HRV indices during the UKT. Among the HRV indices calculated, RMSSD and HF were significantly higher when they ingested the milk protein drink compared with the placebo drink (*p* = 0.019 and *p* = 0.029, respectively), as shown in Figure 4. Large effect size was found in favor of the acidified milk protein drink condition (*r* = 0.54 and *r* = 0.50, respectively). Heart rate, IBI, the standard deviation of IBIs, LF and LF/HF were similar between conditions, as was body temperature.

## 4. Discussion

The present study demonstrated that subjects improved in cognitive performance when they consumed an acidified milk protein drink containing 16 g of milk proteins compared with an isoenergetic placebo drink. In addition, we confirmed that subjects, when they consumed the milk protein drink, also showed an increased HRV, which has been recently suggested to correlate with cognitive function. To the best of our knowledge, this is the first study to show that a single administration of a protein supplement is associated with improved cognitive performance and increased HRV in comparison with isoenergetic-fed controls.

Recent reviews have suggested that breakfast consumption may acutely improve memory, attention, motor and executive function, although the contribution of macronutrients on cognitive function is inconclusive [32]. Our result shows a significant increase in the number of correct answers in the UKT when subjects consumed an acidified milk protein drink. The UKT consists of simple mental arithmetic and handwriting and is used to measure cognitive task performance [33,34,35,36]. Mental arithmetic requires the involvement of several cognitive domains including short-term memory and sustained attention [37]. Handwriting is also a complex perceptual–motor skill [38]. On the other hand, the subjects’ scores on the Stroop test did not improve after they drank the protein drink. This might be partly because the Stroop test results were measured inappropriately. In this study, we used the paper-based version of the Stroop test repeatedly throughout the test sessions, such that subjects answered the same questions repeatedly within the same day; the subjects may have memorized the questions as a result. The Stroop test measures executive function, which would involve higher and more complex cognitive processes [39]. Therefore, it is also possible that consumption of milk protein drinks may not have an acute effect on executive function. In addition, we found no differences in the changes in subjective feeling between drink conditions. Thus, we concluded that the ingestion of the acidified milk protein drink must have improved cognitive performance without changing the subjects’ feelings.

The body temperature was similar between the drink conditions. While protein feeding has been observed to have a higher thermic effect compared with other macronutrients [40], it is unlikely that the 16 g of protein in the test drink was enough to increase the peripheral body temperature of the subjects in this study.

The HF and RMSSD of HRV during the UKT were higher when the subjects ingested the acidified milk protein drink compared with the control drink. It is assumed that both RMSSD and HF, which are the indices of the time domain analysis and frequency domain analysis of HRV, respectively, indicate the vagal modulation of the cardiac function. Thus, the increases in RMSSD and HF should indicate that ingestion of the milk protein drink was associated with stronger vagal activity. However, HRV may be more than just an indicator of simple cardiac and autonomic function [26,41,42]. Growing evidence suggests that a higher vagally mediated HRV is associated with greater cognitive performance [43,44,45,46,47,48]. To account for this relationship, a neurovisceral integration model has been proposed [49,50]. The neurovisceral integration model explains how multilevel neural control can adaptively coordinate cognitive and autonomic response depending on one’s current goals and context (e.g., a particular cognitive task, the state of the body, and the external world). The model explains, in addition, that the higher HRV might be the index for greater prefrontal level control, which is sensitive to goals and context and required to successfully perform demanding cognitive/attentional tasks. The increase in HRV after intake of the acidified milk protein drink may have correlated with greater prefrontal level control, which in turn would have led to the improved UKT scores.

The mechanism for the relationship of milk protein drinks with improved cognitive performance, compared with the placebo, remains to be elucidated. One hypothesis involves the distinct dynamics of neurotransmitter precursors after ingestion of the different nutrients, which would influence brain function. Ingestion of either proteins or carbohydrates modifies the uptake of tryptophan and tyrosine into the brain and the conversion to serotonin and catecholamines, respectively [51,52]. In addition, a recent review described how the manipulation of tryptophan could affect the gut–brain axis, which could in turn affect cognitive function [53]. Previous animal study has shown that ingestion of acidified milk may lead to greater post-exercise muscle protein synthesis through a greater increase of essential amino acids in plasma compared with bovine skim milk [22]. Thus, the ingestion of milk protein drinks may have affected cognitive performance through changes in neurotransmitter precursor concentrations, although changes in amino acids levels after consumption of the acidified milk should be investigated in humans. Another hypothesis is that the peptides generated from acidified milk protein might have affected cognitive function. Nakamura et al. reported that ingestion of 10 g of casein hydrolysates was associated with improved cognitive performance in healthy young men, with increased HRV also observed [54,55]. In the acidification process, stabilizers and dispersants are added to prevent aggregation of milk proteins [22], apparently leading to quick digestion of the milk protein, as shown in the greater increase in amino acids in the blood after the acidified milk protein consumption. Digestion of the acidified milk protein test drink in our study may have generated peptides similar to those in the casein hydrolysates. Further study comparing the acidified milk protein with bovine milk protein is necessary to understand the relationship of milk protein acidification with cognitive performance and HRV.

However, there are limitations in determining the detailed mechanism for the changes in cognitive function observed in this study. The difference in the amount of glucose, rather than the proteins, between the 2 test drinks could have affected cognitive performance. An ‘inverted U’-shaped dose-response curve was observed for the relationship of plasma glucose level with cognition [56]. Acute glucose fluctuations may impair cognitive performance [57] and a stable metabolic condition should, therefore, improve cognitive performance [58]. In addition, the 2 test drinks differ in other components as well as proteins and carbohydrate constituents: namely, the stabilizers and acidifier for the acidification procedure, and the sweeteners and flavors, which were added so the test drinks would have a similar taste and appearance. These components might have affected cognitive performance and autonomic activity in this study. Some factors involved in cognition were not considered in this study. Individual differences in fasting glucose appear to be relevant for cognition [59] and baseline glucoregulation has been demonstrated to moderate postprandial cognition [18] even in healthy young adults. Links between obesity and cognitive function have also been reported in young adults [60]. Further studies are necessary to clarify the mechanism by which the acidified milk protein drink improved the subjects’ cognitive performance and also increased their HRV.

## 5. Conclusions

Intake of a single dose of an acidified milk protein drink containing 16 g of protein was associated with a significant increase in performance of a serial arithmetic task in healthy young adults. In addition, the drink was associated with a significant increase in vagally-mediated HRV indices related to cognitive performance. These results suggest that the acidified milk protein drink may improve cognitive performance, with the possible involvement of physiological systems that regulate cognition. Even though the mechanism for this improvement in cognitive performance needs clarification, as does the effect of the acidification process, it is an important finding that consuming an adequate amount of protein within a single meal can improve cognitive performance.

## Figures and Tables

**Table 1:** Composition of the 430 mL of test drinks.

	Acidified Milk Protein Drink	Placebo Drink
Basic ingredients (%) ^1^		
Milk protein ^2^	4.4	
Glucose ^3^	4.6	8.5
Trehalose ^4^	1.0	1.0
Fermented cellulose ^5^	0.05	
Pectin ^6^	0.1	
Soybean polysaccharide ^7^	0.45	0.45
Citric acid ^8^	0.32	0.13
Malic acid ^9^	0.27	0.11
Sodium citrate ^10^		0.27
Vitamin B6 ^11^	0.00019	0.00019
Water	88.7	89.0
Nutrient (g)		
Carbohydrate	28	42
Protein	16	0
Fat	0.3	0.9
Energy (kcal)	176	176

^1^ Other than basic ingredients, both drinks contained 0.05% mixture of flavors, and the milk protein drink contained a mixture of sweeteners (sucralose and acesulfame K) at a concentration of 0.012% to obtain a similar taste and flavor. The placebo drink contained emulsified flavor at a concentration of 0.5% to obtain a cloudy color similar to that of the milk protein drink. The milk protein drink had a defoaming agent added to a concentration of 0.05% to dissolve the milk protein. Both drinks were adjusted to have the same pH; ^2^ milk protein concentrate-80 (Idaho Milk Products, Inc., Jerome, ID, USA); ^3^ glucose (San-ei Sucrochemical Co., Ltd., Aichi, Japan); ^4^ trehalose (Hayashibara Co., Ltd., Okayama, Japan); ^5^ fermented cellulose (San-Ei Gen F.F.I., Inc., Tokyo, Japan); ^6^ pectin AYD5110SB (Unitec Foods Co., Ltd., Tokyo, Japan); ^7^ soybean polysaccharide (San-Ei Gen F.F.I., Inc., Tokyo, Japan); ^8^ citric acid (Iwata Chemical Co., Ltd., Shizuoka, Japan); ^9^ DL-malic acid (Fuso Chemical Co., Ltd., Osaka, Japan); ^10^ risodium citrate (Iwata Chemical Co., Ltd., Shizuoka, Japan); ^11^ pyridoxine hydrochloride (Kongo Yakuhin Co., Ltd., Toyama, Japan).
